# Intrinsic generation time of the SARS-CoV-2 Omicron variant: An observational study of household transmission

**DOI:** 10.1016/j.lanepe.2022.100446

**Published:** 2022-07-01

**Authors:** Mattia Manica, Alfredo De Bellis, Giorgio Guzzetta, Pamela Mancuso, Massimo Vicentini, Francesco Venturelli, Alessandro Zerbini, Eufemia Bisaccia, Maria Litvinova, Francesco Menegale, Carla Molina Grané, Piero Poletti, Valentina Marziano, Agnese Zardini, Valeria d'Andrea, Filippo Trentini, Antonino Bella, Flavia Riccardo, Patrizio Pezzotti, Marco Ajelli, Paolo Giorgi Rossi, Stefano Merler, the Reggio Emilia COVID-19 Working Group

**Affiliations:** a Center for Health Emergencies, Fondazione Bruno Kessler, Trento, Italy; b Department of Mathematics, University of Trento, Trento, Italy; c Epidemiology Unit, Azienda Unità Sanitaria Locale – IRCCS di Reggio Emilia, Reggio Emilia, Italy; d Public Health Department, Azienda Unità Sanitaria Locale – IRCCS di Reggio Emilia, Reggio Emilia, Italy; e Unit of Clinical Immunology, Allergy and Advanced Biotechnologies, Azienda Unità Sanitaria Locale – IRCCS di Reggio Emilia, Italy; f Laboratory for Computational Epidemiology and Public Health, Department of Epidemiology and Biostatistics, Indiana University School of Public Health, Bloomington, IN, USA; g Dondena Centre for Research on Social Dynamics and Public Policy, Bocconi University, Milan, Italy; h Dipartimento di Malattie Infettive, Istituto Superiore di Sanità, Rome, Italy

**Keywords:** Generation time, Omicron, SARS-CoV-2, COVID-19, Bayesian inference, Contact tracing

## Abstract

**Background:**

Starting from the final months of 2021, the SARS-CoV-2 Omicron variant expanded globally, swiftly replacing Delta, the variant that was dominant at the time. Many uncertainties remain about the epidemiology of Omicron; here, we aim to estimate its generation time.

**Methods:**

We used a Bayesian approach to analyze 23,122 SARS-CoV-2 infected individuals clustered in 8903 households as determined from contact tracing operations in Reggio Emilia, Italy, throughout January 2022. We estimated the distribution of the intrinsic generation time (the time between the infection dates of an infector and its secondary cases in a fully susceptible population), realized household generation time, realized serial interval (time between symptom onset of an infector and its secondary cases), and contribution of pre-symptomatic transmission.

**Findings:**

We estimated a mean intrinsic generation time of 6.84 days (95% credible intervals, CrI, 5.72–8.60), and a mean realized household generation time of 3.59 days (95%CrI: 3.55–3.60). The household serial interval was 2.38 days (95%CrI 2.30–2.47) with about 51% (95%CrI 45–56%) of infections caused by symptomatic individuals being generated before symptom onset.

**Interpretation:**

These results indicate that the intrinsic generation time of the SARS-CoV-2 Omicron variant might not have shortened as compared to previous estimates on ancestral lineages, Alpha and Delta, in the same geographic setting. Like for previous lineages, pre-symptomatic transmission appears to play a key role for Omicron transmission. Estimates in this study may be useful to design quarantine, isolation and contact tracing protocols and to support surveillance (e.g., for the accurate computation of reproduction numbers).

**Funding:**

The study was partially funded by EU grant 874850 MOOD.

**Research in context**

**Evidence before this study**

We searched PubMed, Google Scholar, and medRxiv for manuscripts in English from database inception to May 4, 2022, with the query (“COVID-19” OR “SARS-CoV-2”) AND (“generation time” OR “generation interval”) AND (“Omicron” OR “B.1.529”). We identified two preprints attempting to infer the generation time of Omicron from the trajectories of variant frequencies at the population level in Denmark and the growth rates of Omicron and Delta in the United Kingdom, respectively. Both studies suggest a significant reduction in the mean generation time of Omicron, estimated at values ranging between 50% and 80% the generation time of Delta. We also found one preprint analyzing 43 infector-infectee pairs from contact tracing data in Hong Kong that estimated a mean realized generation time of 2.38 days (95% confidence interval 2.01-2.80). This estimate was obtained under very strict control measures (population-wide screenings and quarantine imposed to both contacts and contacts of contacts) that are known to reduce the realized generation time.

**Added value of this study**

Using a Bayesian inference model for the reconstruction of the transmission links, we estimated the distribution of the generation time from over 23,000 Omicron infections clustered in about 9,000 households, and diagnosed in the province of Reggio Emilia, Italy, throughout January 2022. Although we found a reduced realized generation time in households for Omicron with respect to equivalent results for Alpha and Delta in the same population (likely due to increased transmissibility), we found no difference in the intrinsic generation time (corresponding to the generation time that would be measured in a fully susceptible population) of Omicron compared to estimates of previous lineages in Italy.

**Implications of all the available evidence**

These results suggest that the intrinsic time elapsing between successive infections has not significantly shortened for the Omicron variant compared to previous variants and provide insights for further characterizing the transmission patterns of the SARS-CoV-2 Omicron variant and for policy evaluation.

Alt-text: Unlabelled box

## Introduction

The SARS-CoV-2 Omicron variant emerged at the end of 2021 and was able to completely replace the dominant variant Delta with a swiftness that was unprecedented compared to previously emerged lineages.^1^ The exceptional fitness of Omicron is likely due to a combination of competitive advantages, including an increased transmissibility,^2^ and the ability to escape the immune response both from natural infection and vaccination.^3^^,^^4^ The takeover of Omicron was accompanied by peak incidence values that were several times higher than the previous record in most countries of the world.

Early after its emergence, studies have indicated that Omicron may have shorter incubation period^5^, ^6^, ^7^ and serial interval.^5^, ^6^^,^^8^ This observation, although based on preliminary evidence, has led many countries to abbreviate the duration of quarantine and isolation^9^^,^^10^ in the attempt to contain the negative effect of high simultaneous absenteeism on the economy. However, whether the reduction in incubation periods and observed serial intervals reflects a reduction of the generation time, i.e., the time that elapses between the infection episode of an infector and of its infectee, is still to be determined. The generation time is an important parameter for monitoring and modeling infectious diseases. For example, if quarantine and isolation mandates are based on the generation time (as a proxy of infectiousness over time), an underestimation of this parameter would imply an early release of individuals when they still have a high probability to infect others, resulting in a reduced effectiveness of the intervention; on the other hand, an overestimation would imply an unnecessarily lengthy limitation of individual freedoms and increased absenteeism from school and workplaces, with impacts on the economy and the society. Biased estimates of the generation time also impact on the accuracy of the estimate of the net reproduction number, which is a key quantity for epidemiological surveillance, often used also as a parameter for deciding interventions. Finally, the generation time is largely used in scientific research (including mathematical modeling), for applications such as evaluating the effectiveness of interventions, understanding epidemiological dynamics, reconstructing transmission chains.

A direct measure of the generation time cannot be obtained empirically because the infection episodes in a chain of transmission are generally unobservable. Even when some certainty can be attributed to the dates of infection for pairs of infectors-infectee via detailed epidemiological investigation, the observed (“realized”) generation times may be biased by the specific transmissibility conditions, including the structure of the study population's contact network, individual behaviors, environmental determinants, and control measures put in place.^11^ For example, it is known that the generation time realized in households is remarkably shortened with respect to the one observed in the general community, due to the depletion of susceptible individuals and the competition of simultaneously infectious individuals to find susceptible household members to infect.^11^ In contrast to the realized generation time, occurring in a realistic network of contacts, the “intrinsic” generation time represents the generation time that would be observed in a fully susceptible, homogenously mixed population.^12^ The intrinsic generation time is therefore less dependent on the specific conditions of the epidemiological setting from which it is inferred but must be estimated with the use of quantitative inference techniques.

In this study, we collected the data for 23,122 SARS-CoV-2 infected individuals clustered in 8,903 households as determined from contact tracing operations in Reggio Emilia, Italy, between January 1^st^ and January 31^st^, 2022. We then leveraged a Bayesian inference approach to estimate the distribution of the generation time (both intrinsic and realized), realized household serial interval, and contribution of pre-symptomatic transmission for SARS-CoV-2 Omicron variant.

## Methods

### Data

To mitigate the spread of SARS-CoV-2, contact tracing activities were carried out in the province of Reggio Emilia, Italy, throughout the duration of the pandemic. Identified COVID-19 cases occurring in the province were confirmed via a Polymerase Chain Reaction (PCR) assay, reported in real time to the public health service of the Reggio Emilia local health authority, and isolated at home until either a negative PCR test result or 21 days were elapsed. During the study period (from January 1st to January 31st, 2022), both antigenic positive tests and PCR tests were considered for COVID-19 diagnosis. Household members were tested and quarantined at home. After 5 days (for individuals who were fully vaccinated more than 4 months before the date of contact) or 10 days (for unvaccinated individuals), a PCR test was performed and in the case of a negative result their quarantine was ended. If the test was not taken, household members were quarantined for 14 days, as per national guidelines.^13^ Contacts with a booster dose or complete vaccination cycle or recovered from a previous SARS-CoV-2 infection in the 4 months before the date of contact were not subject to quarantine and were tested only upon development of symptoms. Compliance with at least one of the tests proposed by the public health service was 97.4% during the study period.

Data on test results and symptom onset dates (if applicable) for all identified cases and their contacts were linked to individual records on vaccination history (first, second, and booster dose). Appropriate data quality checks were conducted in strict collaboration with the Reggio Emilia local health authority to minimize missing information and accurately define household clusters. A household cluster was defined as households with at least two positive individuals with a diagnosis spaced less than 14 days apart.

A random sample of new diagnoses was characterized for the viral variants of SARS-CoV-2. Viral RNA was extracted from nasopharyngeal swab and specimens were screened by a commercial multiplex Real-Time PCR assay (SARS-CoV-2 Variants I and II Allplex kit, Seegene; Seoul, South Korea) detecting L452R, W152C, K417T, K417N, E484Q, E484K, and N501Y mutations and HV69/70 deletion, and able to identify SARS-CoV-2 variants Alpha, Beta, Gamma, Delta, Epsilon, Omicron BA.1 and Omicron BA.2.

### Ethics

The collection of data used for this manuscript (surveillance and contact tracing data) is compulsory in Italy according to national laws on infectious diseases. The COVID-19 Italian National Working group on Bioethics has stated that consensus for the collection of this data in the context of the COVID-19 emergency is not mandatory (Rapporto ISS COVID-19 n. 34/2020), based on Guideline 12 of the WHO on ethical issues in public health surveillance. The legal ordinance n. 640 of February 28 2020, explicitly declares Istituto Superiore di Sanità as entitled to collect data for COVID-19 surveillance and contact tracing and that such data can be used and shared, upon anonymization, to advance scientific knowledge on this new disease.

### Estimation of the incubation period

The incubation period was estimated using data from a superspreading event occurred on November 26, 2021, in Norway, where 81 individuals were infected with the Omicron variant at a company's Christmas dinner, 80 of which became symptomatic.^5^ We fitted a gamma distribution to the empirical distribution of incubation periods, and a nonparametric bootstrap resampling to assess uncertainty in the parameters (see Appendix for details).

### Estimation of generation time and serial interval

For the estimation of the generation time, we selected only household clusters for which all dates of diagnosis were included between January 1 and January 31, 2022. To reduce the possibility of missed diagnoses in the households, we further selected households for which undiagnosed members had at least one negative test result. We extended a Bayesian inference model for the reconstruction of transmission links in households.^14^^,^^15^ The model exploits the temporal information on SARS-CoV-2 infections recorded in the dataset to probabilistically identify, for every infection, the likely source of infection (from outside the household or from a specific household member). Parameters for the generation time, which we assume to be gamma-distributed, are simultaneously calibrated via a Markov Chain Monte Carlo approach where the likelihood of the observed data is defined mechanistically through the computation of the force of infection to which all individuals are subject over time. The force of infection includes information on the temporal incidence of cases in the general population, on the date of infection and vaccination history for any individual and on previous infection from other variants. For each symptomatic case, the date of infection was imputed by subtracting the time of symptom onset by a randomly sampled incubation period from the estimated distribution. The imputed dates of infection for symptomatic individuals defined a distribution of delays between infection and diagnosis (diagnostic delay distribution), which was used to impute the date of infection of asymptomatic individuals starting from their date of diagnosis. For both symptomatic and asymptomatic individuals, we set to zero the probability of imputed dates of infection that preceded the latest negative test result. The sampling of infection dates was repeated 100 times and the Bayesian model was re-calibrated on each resampling. Credible intervals (CrI) for the estimated parameters were obtained from the 95% percentile of the resulting pooled distributions.

**Figure 1:**
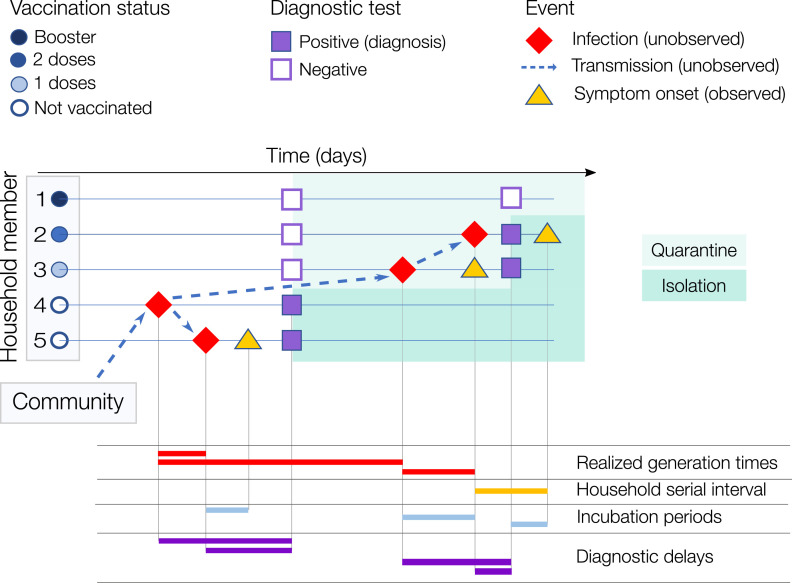
Illustrative example of a household cluster. A household with 5 members, of which #4 (asymptomatic) was infected outside the household (in the general community) and then transmitted to cases #5 and #3 (both symptomatic). Case #3 infected #2 while #1 remained uninfected. #3, #2 and #1 were vaccinated with 1 dose, 2 doses, and 2 doses + booster respectively. In the bottom part of the figure, we show examples of the temporal intervals of interest for this work. Note that for the household serial interval and the realized household generation time, the source of infection (whether from outside the household or from a household member, and, in the latter case, which household member) is also unobserved and needs to be probabilistically reconstructed. The intrinsic generation time is not displayed as it represents the distribution of generation times among infections occurring in the general population in a fully susceptible population.^11^

The inferred transmission links allowed us to estimate, in addition to parameters of the intrinsic generation time, the distribution of the realized household generation time. We also estimated the distribution of the household serial interval from the difference of symptom onset dates in each infector-infectee pair (as inferred by the model) where both are symptomatic. Figure 1 schematizes a potential household cluster, with an indication for each individual of the dates of infection, symptom onset, diagnosis and negative tests, and summarizes the relevant quantities for the purpose of the study. A full description of the Bayesian inference model is available in the Appendix.

### Sensitivity analyses

To test the robustness of our results, we conducted an extensive set of sensitivity analyses where we considered: a) the subset of 380 households (1,127 cases in total) for which a case was genotyped as Omicron; b) the subset of 1,148 households (2,770 cases in total) for which all individuals were unvaccinated; c) the estimated incubation period of the Delta variant (mean: 4.5 days; standard deviation: 2.1 days)^15^ to reassign the imputed infectious dates (baseline: Omicron variant with mean 3.5 days and standard deviation: 1.2 days); d) a diagnostic delay for asymptomatic cases that was 50% longer than that for symptomatic cases, implemented by increasing the shape of the gamma distribution by 50% (mean: 7.58 days; standard deviation: 1.61 days); e) similar to d), but implemented by changing the scale of the gamma distribution by 50% (mean: 7.58 days; standard deviation: 1.97 days); f) the possibility of false negatives for negative test results when imputing infection dates; g) a halved transmissibility for asymptomatic individuals (baseline: equal to symptomatic individuals); h) a halved transmissibility for vaccinated individuals (baseline: equal to unvaccinated individuals); i) a scenario in which any effort to quarantine positive cases would not impact the force of infection from outside the household, which corresponds to the extreme case where there is 0% compliance to the policy (baseline: 100% compliance); j) that previous infection from other variants provides no cross-protection against Omicron (baseline: 56% cross-protection^16^).

### Role of the funding source

The sponsor of the study had no role in study design, data collection, data analysis, data interpretation, or writing of the report. The corresponding author had full access to all the data in the study and had final responsibility for the decision to submit for publication.

## Results

The study considered 8,903 households with mean size of 2.7 (standard deviation: 1.05, 95% quantile: 2–5) and a total of 23,122 diagnosed infections diagnosed between January 1st and January 31st, 2022. Of these, 9,637 (41.7%) were symptomatic and 11,980 (51.8%) were among women (see Table 1). A significant proportion of cases included in the study were unvaccinated (7,164, corresponding to 31%) and only 4,651 (20%) had received a booster dose before the end of the study period, compared to national statistics on the vaccination status of the Italian population on January 31 (17% unvaccinated, 56% with a booster dose^17^). Further descriptive statistics on the data are provided in Table 1.

**Table 1:** Descriptive statistics of SARS-CoV-2 cases in the household dataset.

Period	JANUARY 1 – 31, 2022
Number of cases	23,122
Clinical outcome (%):	
Symptomatic	9,637 (41.7%)
Asymptomatic	13,465 (58.3%)
Gender (%):	
Male (%)	11,142 (48.2%)
Female (%)	11,980 (51.8%)
Age group (%):	
0-15 years old	6,138 (25.6%)
16-44 years old	9,396 (39.1%)
45-64 years old	5,952 (24.8%)
65+ years old	2,532 (10.5%)
Vaccination status at the end of the period (%):	
1 dose	1,132 (4.9%)
2 doses	10,175 (44.0%)
3 doses	4,651 (20.1%)
None	7,164 (31.0%)
Number of households	8,903
Mean household size (95% quantile)	2.70 (2 – 5)

From the analysis of symptom onset data of 74 individuals participating to a superspreading event in Norway,^5^ we estimated a gamma-distributed incubation period of mean: 3.49 days (standard deviation: 1.20 days, 95%CrI: 3.19–3.77), see Table 2.

**Table 2:** Estimates for the incubation period, diagnostic delay, intrinsic and realized generation time, and household serial intervals of the SARS-CoV-2 Omicron variant.

INCUBATION PERIOD	mean (95%CrI) [days]	3.49 (3.19-3.77)
	95% quantile of the mean distribution [days]	2-6
	shape mean (95%CrI)	8.50 (6.14-13.20)
	scale mean (95%CrI)	0.41 (0.25-0.68)
	standard deviation of the mean distribution [days]	1.20
DIAGNOSTIC DELAY FOR SYMPTOMATIC INDIVIDUALS	mean (95% quantile) [days]	5.05 (3-7)
	standard deviation [days]	1.31
INTRINSIC GENERATION TIME	mean (95%CrI) [days]	6.84 (5.72-8.60)
	95% quantile of the mean distribution [days]	1-17
	shape mean (95%CrI)	2.39 (2.01-3.34)
	scale mean (95%CrI)	2.95 (1.81-4.25)
	standard deviation of the mean distribution [days]	4.48
REALIZED HOUSEHOLD GENERATION TIME	mean (95%CrI) [days]	3.59 (3.55-3.60)
HOUSEHOLD SERIAL INTERVAL	mean (95%CrI) [days]	2.38 (2.30-2.47)
PRE-SYMPTOMATIC TRANSMISSION	mean (95%CrI) [%]	51.1 (45.5-55.7)

Reported parameters of shape and scale for the incubation period and intrinsic generation time refer to a gamma distribution. Estimates of the incubation period are derived from the analysis of 80 participants to a single superspreading event in Norway. Data taken from Brandal et al.^5^

**Figure 2:**
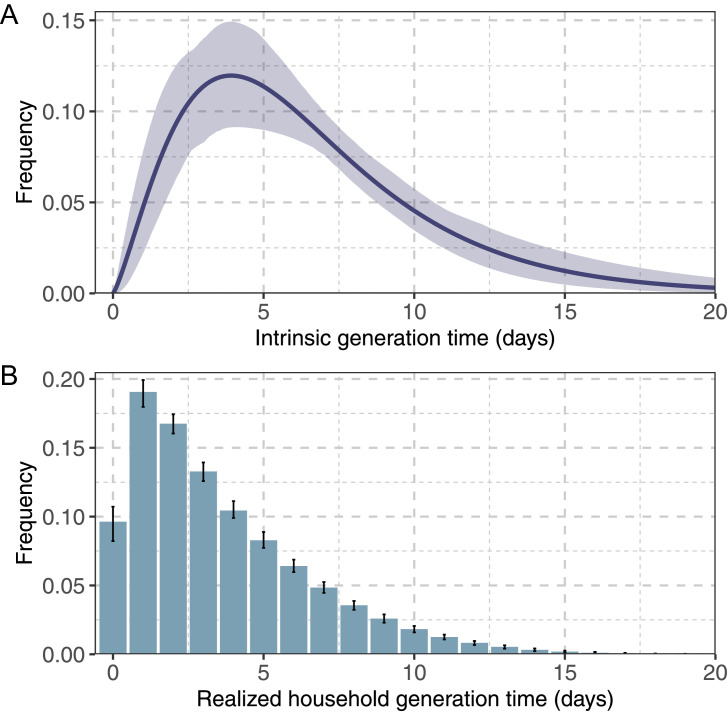
Estimates of the generation time for the Omicron variant. A) Distribution of the intrinsic generation time; solid line: mean estimate; shaded area: 95% CrI; B) Distribution of the realized household generation time; bars: mean estimate; vertical lines: 95% CrI.

By leveraging the estimated distribution of incubation period, we estimated a distribution of delays between infection and diagnosis having a mean of 5.05 days (standard deviation: 1.31 days, 95% quantile: 3–7 days) (Table 2) for symptomatic subjects. By applying the Bayesian inference model, we estimated a mean intrinsic generation time of 6.84 days (95% CrI of the mean: 5.72–8.60 days) and a mean realized household generation time of 3.59 days (95% CrI of the mean: 3.55–3.60 days) (Figure 2 and Table 2).

**Figure 3:**
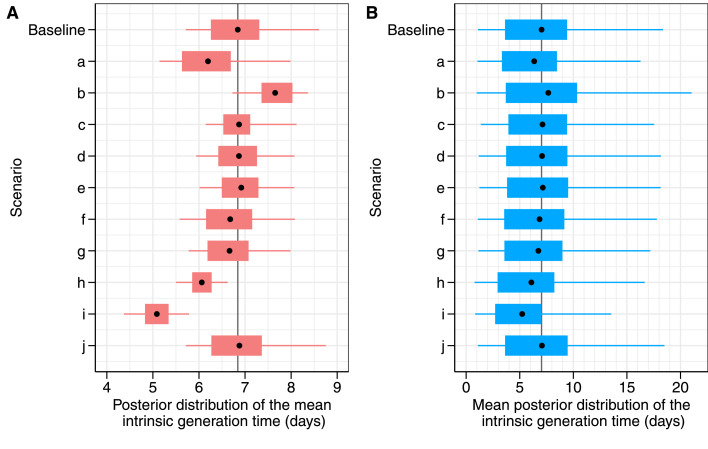
Estimates of generation times for the Omicron variant under different sensitivity analyses. A) Posterior distributions of the mean intrinsic generation time; B) Mean distributions of the intrinsic generation time. Point: mean value; box: interquantile range; whiskers: 95% CrI. The labels on the y-axis represent the performed sensitivity analysis to evaluate the robustness of baseline model results against different model assumptions where we consider: a) only households genotyped as Omicron; b) only household composed of unvaccinated individuals; c) an incubation period for Omicron with the same distribution as previous estimates for Delta (mean: 4.5 days; standard deviation: 2.1 days)^18^; d) a prolonged diagnostic delay for asymptomatic individuals (mean: 7.58 days, standard deviation: 1.61 days); e) a prolonged diagnostic delay for asymptomatic individuals (mean: 7.58 days, standard deviation: 1.97 days); f) the possibility of false negative tests; g) a halved transmissibility for asymptomatic individuals; h) a halved transmissibility for vaccinated individuals; i) a scenario where any effort to quarantine positive cases would not impact the force of infection from outside the household; j) previous infection from other variants provides no cross-immunity against Omicron infection.

The robustness of these estimates was tested against several sensitivity analyses regarding selected subsets of the sample (sensitivity analyses a and b), alternative imputation methods for infection times (c-f) and alternative modelling assumptions (g-j; see Appendix for full details). All sensitivity analyses yielded comparable results with respect to the distribution of the intrinsic generation time (Figure 3), with 95% confidence intervals broadly overlapping with the baseline estimate, except for a significantly shorter mean estimate (5.09 days) obtained when assuming no compliance to household quarantines. The longest mean realized household generation time (3.96 days) was estimated under the assumption of an incubation period equal to the one estimated for Delta, while the shortest (3.24 days) was estimated when considering only unvaccinated individuals.

The mean household serial interval in the baseline analysis was 2.38 days (95%CrI of the mean: 2.30–2.47 days), with 51.1% (95%CrI: 45.5–55.7%) of transmission episodes being pre-symptomatic (i.e., secondary cases transmitted by cases who would develop symptoms after the transmission episode). The mean household serial intervals estimated in sensitivity analysis ranged between 1.89 and 2.38 days (Appendix), while the mean proportion of pre-symptomatic transmission ranged between 51% and 59%, comparable to the baseline estimate.

## Discussion

We analyzed comprehensive data collected during contact tracing activities on over 23,000 SARS-CoV-2 cases distributed in about 9,000 households from the province of Reggio Emilia, Italy, between January 1 and 31, 2022. Our estimate of the mean generation time (mean: 6.8 days) is compatible with previous estimates for ancestral lineages^18^ (including a previous estimate for Italy of 6.7 days^19^). Existing estimates for Alpha and Delta were in the same range in a study similar to the present one on the same study population in Italy (6.0 and 6.6 days respectively^15^). We also found a mean household serial interval of 2.38 days, shorter than previous estimates for Delta of 2.56 on a similar study population.^15^ Available studies have suggested a shorter generation time of Omicron (between 50% and 80% the one of Delta) using population-level data on the growth rate of Omicron relative to Delta in Denmark^20^ and United Kingdom.^21^ An analysis of 43 infector-infectee pairs from contact tracing data in Hong Kong^22^ estimated a mean realized generation time of 2.38 days (95% confidence interval 2.01–2.80) under very strict control measures (population-wide screenings and quarantine imposed to both contacts and contacts of contacts) that are known to reduce the realized generation time. Generalization of epidemiological estimates to different geographic contexts and conditions (and therefore their direct comparison) always needs to be made with caution. We believe that the provided estimate may be representative for places with similar socio-economic conditions and housing structure. For example, the mean number of residents per housing unit is similar between Reggio Emilia and the rest of Italy (Reggio Emilia: 2.44; Italy: 2.42; national range across NUTS1 aggregations: 2.31–2.67) and similarly for the mean number of residents per room (Reggio Emilia: 0.55; Italy: 0.57; national range across NUTS1 aggregations: 0.54–0.63).^23^ However, other factors such as seasonality in transmission, mitigation measures, testing efficiency, or the progression of the vaccination campaign may make direct comparison of estimates performed at different times problematic, even when they come from the same study population.

The result that the intrinsic generation time of the Omicron variant in Italy is not significantly shorter than previous lineages may be surprising with respect to the intuition suggested by repeated observations of shorter incubation periods^5^, ^6^, ^7^ and serial intervals^5^^,^^6^^,^^8^ (the latter also confirmed by this study). Realized serial intervals in households and other small-population settings such as schools, workplaces, hospital wards and nursing homes, may be a biased proxy for the intrinsic generation time since they depend strongly on the epidemiological conditions of the study population^11^^,^^12^; in particular, they tend to be shorter when transmissibility is higher (as in the case of the Omicron variant) because the competition for susceptible individuals is stronger.^12^ For example, simulating transmission in households through a simple generative model where we imposed a mean generation time of 6.9 days, the mean realized generation time in households turned out to be 4.7 days because of this competition effect (see Appendix). On the other hand, the incubation period only reflects a clinical condition (development of symptoms) that is known to be poorly correlated to infectiousness for COVID-19, given the large proportion of infections transmitted by asymptomatic and pre-symptomatic individuals. The duration of viral shedding is likely a better biological proxy of the intrinsic generation time, as it is more closely related to the intrinsic infectiousness of an infected individual.^24^ Several studies have found a similar duration of viral shedding for Omicron and other variants,^25^, ^26^, ^27^ in agreement with the conclusions from our study.

A main strength of this work consists in the very large population-based dataset that comprehensively covers household clusters observed in the province of Reggio Emilia. Public health officials made efforts to have high compliance to testing policies (97.4% of individuals who were offered a test accepted at least once), including testing all household members of cases at the date of the first diagnosis in the household. However, the following limitations should be taken into consideration to interpret our results. First, the main analysis relied on cases diagnosed during the study period. The prevalence of the Omicron variant in the region (Emilia-Romagna) was 80% among infections diagnosed on January 3, 2022, rising to 99% among infections diagnosed on January 17 and 31^28^; more specifically, over 97% of all infections diagnosed in Italy on January 31 were classified as Omicron sublineage BA.1.^28^ Therefore, it cannot be excluded that a minority of cases in our sample belonged to other variants. However, a sensitivity analysis performed on 380 households (1,127 cases) for which a case was genotyped as Omicron yielded compatible results. Second, the model relies on assumptions for the dates of infection of infected individuals; nonetheless, estimates were substantially robust with respect to different imputations of the dates of infection (i.e., by using the incubation period estimated for Delta, different distributions of the diagnostic delay for asymptomatic individuals, and allowing the possibility of false negative test results, see Figure 3 and Appendix). The same intrinsic limitation of the unobservability of infection times is shared by all transmission chain reconstruction models, but there are now several examples where these models have been proven to correctly identify the transmission dynamics of infectious outbreaks.^29^^,^^30^ It is also important to stress that for traced contacts, the detection of symptoms was done at the time of diagnosis; as such, if symptoms appeared in the days following the positive swab, the infected individual was recorded as asymptomatic. Another specific limitation is that compliance to quarantine protocols is unknown; we assumed 100%, i.e., that household members quarantined after diagnosis of another member could only be infected within the household. If compliance was imperfect in the considered population, infected household members may have contracted the infection from the general community, especially considering the very high incidence observed in January 2022 in Italy. A sensitivity analysis where quarantines of household members are not considered (i.e., 0% compliance) yielded a significantly shorter mean intrinsic generation time (5.1 vs 6.8 days), because in this case longer generation times that were attributed to potential household infectors in the baseline analysis are preferentially attributed to an importation from the general community. As a result, the mean estimate of the intrinsic generation time may be shorter than the baseline if compliance to quarantine decreased during the period when Omicron was dominant. Lower compliance to quarantine is possible because the lower severity of the Omicron wave in Italy and a general relaxation of control measures induced a lower perception of risk. Considering the ability of Omicron to escape the immune response from vaccination, we assumed no reduction in transmissibility for vaccinated individuals. However, relaxing such assumption by halving the transmissibility for vaccinated individuals yielded shorter yet comparable results in the mean estimate of the intrinsic generation time (6.1 days). Additional sensitivity analysis investigating uncertainties on the transmissibility of asymptomatic individuals, or on the absence of cross-protection from previous infections with other lineages did not affect the main results significantly (Appendix).

In conclusion, we produced robust estimates of the length of the intrinsic generation time for Omicron in Emilia Romagna, Italy, suggesting limited variations with respect to ancestral lineages or variants Alpha and Delta obtained in the same country, and providing useful insights for further characterizing the transmission patterns of the SARS-CoV-2 Omicron variant and for policy evaluation.

## Contributors

G.G., P.Pe., M.A., P.G.R., and S.M. conceived the study. M.M., Ad.B. and G.G. wrote the first draft of the manuscript. M.M., Ad.B., F.M. and C.M.G. wrote the code and performed the analyses. P.M., M.V., F.V., A.Ze., E.B., A.B., F.R., and the members of the Reggio Emilia COVID-19 Working Group collected the epidemiological data. M.M., Ad.B., G.G., F.M., C.M.G., M.L., V.M., P.Po., A.Za., Vd.A., F.T., P.Pe., M.A., P.G.R., and S.M. interpreted results. G.G., P.Pe., M.A., P.G.R. and S.M. supervised the study and accessed and verified the data. All authors read, reviewed, and approved the final version and the submission of the manuscript. The corresponding author had final responsibility for the decision to submit for publication.

## Data sharing statement

Participant data with identifiers and the code for study reproducibility have been uploaded on the Figshare public repository (https://figshare.com/s/c05e5e2b0b36f7e5344e; DOI: 10.6084/m9.figshare.19731583). The data dictionary is included within the README file attached to the code. The data and code will be publicly available with publication.

## Declaration of interests

MA has received research funding from Seqirus. The funding is not related to COVID-19. All other authors declare no conflicts of interest.

## Appendix Supplementary materials

Image, application 1

Image, application 2
