# Supplementary material for: Intrinsic generation time of the SARS-CoV-2 Omicron variant: An observational study of household transmission
Source: Lancet Reg Health Eur. 2022 Jul 1;19:100446. doi: 10.1016/j.lanepe.2022.100446 (PMC9246701; doi:10.1016/j.lanepe.2022.100446)
Supplement: Supplementary file 1 [file mmc1.docx]

**Appendix**

Intrinsic generation time of the SARS-CoV-2 Omicron variant: an observational study of household transmission

Mattia Manica ^a,*^, PhD, Alfredo De Bellis ^a,b,*^, MSc, Giorgio Guzzetta ^a,*^, PhD, Pamela Mancuso ^c^, MSc, Massimo Vicentini ^c^, MSc, Francesco Venturelli ^d^, PhD, Alessandro Zerbini ^e^, PhD, Eufemia Bisaccia ^d^, MD, Maria Litvinova ^f^, PhD, Francesco Menegale ^a,b^, MSc, Carla Molina Grané ^a,b^, MSc, Piero Poletti ^a^, PhD, Valentina Marziano ^a^, PhD, Agnese Zardini ^a^, PhD, Valeria d’Andrea ^a^, PhD, Filippo Trentini ^a,g^, PhD, Antonino Bella ^h^, DStat, Flavia Riccardo ^h^, PhD, Patrizio Pezzotti ^h^, DStat, Marco Ajelli ^f,#^, PhD, Paolo Giorgi Rossi ^c,#^, PhD, Stefano Merler ^a,#,%^, MSc, and the Reggio Emilia COVID-19 Working Group.

1. Center for Health Emergencies, Fondazione Bruno Kessler, Trento, Italy
2. Department of Mathematics, University of Trento, Trento, Italy
3. Epidemiology Unit, Azienda Unità Sanitaria Locale – IRCCS di Reggio Emilia, Reggio Emilia, Italy
4. Public Health Department, Azienda Unità Sanitaria Locale – IRCCS di Reggio Emilia, Reggio Emilia, Italy
5. Unit of Clinical Immunology, Allergy and Advanced Biotechnologies, Azienda Unità Sanitaria Locale – IRCCS di Reggio Emilia, Italy
6. Laboratory for Computational Epidemiology and Public Health, Department of Epidemiology and Biostatistics, Indiana University School of Public Health, Bloomington, IN, USA
7. Dondena Centre for Research on Social Dynamics and Public Policy, Bocconi University, Milan, Italy
8. Dipartimento di Malattie Infettive, Istituto Superiore di Sanità, Rome, Italy

^*^ joint first authors

^#^ joint senior authors

^%^ corresponding author: [merler@fbk.eu](mailto:merler@fbk.eu)

Corresponding author contact:

Stefano Merler

Center for Health Emergencies

Fondazione Bruno Kessler

Via Sommarive 18, 38123

Povo (Trento), Italy

[merler@fbk.eu](mailto:merler@fbk.eu)

The following are members of the Reggio Emilia Covid-19 Working Group: Emanuela Bedeschi, Cinzia Perilli, Nadia Montanari, Francesca Pia Lionetti, Nicoletta Patrignani, Letizia Bartolini, Francesca Roncaglia, Isabella Bisceglia, Valeria Cenacchi, Maria Barbara Braghiroli, Annamaria Pezzarossi

Table of Contents

[1. Estimation of the incubation period 3](#_Toc102665777)

[2. Imputation of dates of infection 3](#_Toc102665778)

[3. Inference of transmission links 4](#_Toc102665779)

[4. Additional results of the baseline model 6](#_Toc102665780)

[*Statistics on reconstructed transmission links* 6](#_Toc102665781)

[*Stability of the attributed source of infection* 7](#_Toc102665782)

[5. Sensitivity Analyses 7](#_Toc102665783)

[*a)* *Only households with confirmed Omicron genotype* 8](#_Toc102665784)

[*b)* *Only household including not vaccinated individuals* 8](#_Toc102665785)

[*c)* *Alternative distribution of the incubation period* 9](#_Toc102665786)

[*d)* *Longer diagnostic delay for asymptomatic cases (increased shape of gamma distribution)* 9](#_Toc102665787)

[*e)* *Longer diagnostic delay for asymptomatic cases (increased scale of gamma distribution)* 9](#_Toc102665788)

[*f)* *False negative test allowed when imputing infection dates* 10](#_Toc102665789)

[*g)* *Reduced transmissibility for asymptomatic individuals* 10](#_Toc102665790)

[*h)* *Reduced transmissibility for vaccinated individuals* 11](#_Toc102665791)

[*i)* *No protection from infection outside the household during quarantine* 11](#_Toc102665792)

[*j)* *No protection from previous infection* 12](#_Toc102665793)

[6. Epidemiological context 12](#_Toc102665794)

[7. Simulation of within-household transmission 13](#_Toc102665831)

[References 13](#_Toc102665832)

1. **Estimation of the incubation period**

We fitted a gamma distribution to the empirical distribution of incubation periods observed during a superspreading event (a company dinner) occurred on November 26, 2021, in Norway [S1]. A nonparametric bootstrap resampling was performed to assess uncertainty in the parameters. In the epidemiological study, 81 of the tracked participants to the dinner developed infection with SARS-CoV-2, and 80 of these were symptomatic. We excluded 6 cases who had symptom onset before the event and considered the empirical distribution of the remaining 74 cases for the baseline estimate. As a sensitivity analysis, we excluded 14 further cases whose samples had not been sequenced (as they could have been infected with a different lineage) and 5 who had come back from travels abroad (of which one from South Africa) within one week before the dinner (as they could have contracted the variant abroad).

The resulting fit for the two analyses are substantially overlapping (Figure S1). We obtained a mean incubation period for Omicron of 3.49 days (standard deviation: 1.20, 95% bootstrap confidence interval, bCI: 3.19-3.77 days) in the baseline analysis (blue line) and of 3.45 days (standard deviation: 1.20 days, 95% bCI: 3.13-3.80) in the sensitivity analysis. The algorithm for the fit was implemented in python (version 3.9.7) using package scipy (version 1.7.1).

**Figure S1. Empirical and fitted distribution of the incubation period for variant Omicron, using data from [S1]**

1. **Imputation of dates of infection**

The task of reconstructing transmission chains must overcome the intrinsic limitation of the unobservability of transmission chains. We use available evidence to probabilistically impute plausible infection dates for all SARS-CoV-2 cases in our dataset. We combine observed dates of symptom onset, diagnosis, and negative test results with available knowledge on incubation periods and the probability of testing positive over time for infected individuals.

First, we impute the dates of infection for all symptomatic cases. Let T_D_ be the date of diagnosis (when the individual tested positive), T_S_ the date of symptom onset, $T_{N}$ the date of the last negative test before symptom onset; we define the following probability P_I_ of being infected on day T_I_:

| $P_{I}(T_{I})=P_{S}\left( T_{s}-T_{I} \right)\cdot H({T_{I}-T}_{N})$ | (Eq. 1) |
| --- | --- |

Where P_S_(*t*) is the probability density function of the incubation period and $H(x)$ is the Heaveside step function (i.e., $H\left( x \right)=0$ for $x<0$ and $H\left( x \right)=1$ for $x\geq0$). For P_S_(*t*) we use the estimate above as a baseline, and an alternative estimate on the delta lineage [S2] as sensitivity analysis (see Section 5c). For each symptomatic case, a time of infection is sampled from P_I_(t) and the date of infection T_I_ is obtained by rounding to the closest integer. The sample is repeated K = 100 times.

For asymptomatic cases, we cannot use the information on the incubation period given that no date of symptom onset is defined. Therefore, we use the imputed dates of infection for symptomatic cases to define a distribution of diagnostic delays P_D_(*x*), defining the probability of being diagnosed after *x* days from infection. An empirical approximation of P_D_(*x*) will be given, for any *x*, by the fraction of all instances across the K stochastic samples for which the diagnostic delay T_R_ = T_D_ – T_I_ is equal to *x*. A gamma function is then fitted to the empirical distribution using a maximum likelihood approach to obtain P_D_(*x*). The infection date of asymptomatic cases can then be sampled from the following probability

| $P_{I}(T_{I})=P_{D}\left( T_{D}-T_{I} \right)\cdot H({T_{I}-T}_{N})$ | (Eq. 2) |
| --- | --- |

assuming that the distribution of diagnostic delays for asymptomatic cases is the same as for symptomatic cases. Figure S2 reports the estimated empirical and fitted distributions of diagnostic delays for variant Omicron.

The algorithm for the imputation of dates of infection was implemented in python (version 3.9.7) using packages numpy (version 1.20.3) and scipy (version 1.7.1).

**Figure S2. Empirical and fitted distribution of the diagnostic delay P_D_ for variant Omicron, estimated from symptomatic cases.** The histogram represents the empirical distribution given the imputed infection times for symptomatic individuals. The curve represents the fitted gamma function.

1. **Inference of transmission links**

The model adopted in this work extends the approach previously proposed in [S2, S3]. We assumed that, at any time t, a susceptible individual j within a household is exposed to a force of infection composed of two components:

| $\lambda_{j}\left( t \right)=\lambda_{j}^{o}\left( t \right)+\lambda_{j}^{h}\left( t \right)$ | (Eq. 3) |
| --- | --- |

Where $\lambda_{j}^{o}\left( t \right)$ represents the force of infection from the general community outside the household, and $\lambda_{j}^{h}\left( t \right)$ represents the one from infected members inside the household.

We define:

| $\lambda_{j}^{O}\left( t \right)=\sum_{z \in0..t} \alpha I\left( z \right)\chi_{j}\left( t \right)\Gamma\left( t-z;a,b \right) q_{j}(t)$ | (Eq. 4) |
| --- | --- |

Where:

- $\alpha$ is a free parameter scaling the transmissibility in the general community;
- $I(z)$ is proportional to the number of newly infected cases at time *z* outside the household of *j*, obtained from epidemic curves by date of symptom onset for the province of Reggio Emilia in the Italian integrated surveillance system [S4, S5];
- $\chi_{j}\left( t \right)$ represents the relative susceptibility of individual *j* and changes over time *t* depending on the dates of vaccination of *j* and on cross-protection given by previous infection with other SARS-CoV-2 lineages;
- $\Gamma\left( t;a,b \right)$ represents the distribution of the intrinsic generation time at day *t* after infection, for which we assumed a discretized Gamma distribution with scale $a$ and shape $b$; in particular, given *g*(*t; a, b*) the continuous Gamma probability distribution, $\Gamma\left( t;a,b \right)=\int_{t}^{t+1} g\left( \tau;a, b \right)d\tau$;
- q_j_(t) is an on/off function that is 0 when the household of *j* is in quarantine and 1 otherwise. For each household, a quarantine of 14 days is started after the first diagnosis and reinstated for a further 14 days every time there is a new diagnosis after the previous quarantine has ended.

In addition, we define $\lambda_{j}^{h}\left( t \right)$ as:

| $\lambda_{j}^{h}\left( t \right)=\sum_{i\in H_{j}} \lambda_{j,i}^{h}(t)=\sum_{i \in H_{j}} \beta\chi_{j}\left( t \right)\Gamma\left( t-T_{I,i};a,b \right)$ | (Eq. 5) |
| --- | --- |

where:

- *i* is an index running over the set *H_j_* of infected household members of individual *j*;
- $\beta$ is a free parameter scaling the transmissibility inside households.

For the relative susceptibility, we assumed that a vaccine dose starts to be protective 14 days after inoculation:

| $\chi_{j}\left( t \right)=\left\{ \begin{aligned} 1 if {t<t}_{v,1}+14 \\ ({1-\chi}^{\left( 1 \right)} e^{-w_{1}\left( t-t_{v,1}-14 \right)}) if t_{v,1}+14\leq t<t_{v,2}+14 \\ ({1-\chi}^{\left( 2 \right)}e^{-w_{2}\left( t-t_{v,2}-14 \right)}) if t_{v,2}+14\leq t<t_{v,3}+14 \\ (1-\chi^{\left( 3 \right)}e^{-w_{3}\left( t-t_{v,3}-14 \right)}) if t\geq t_{v,3}+14 \end{aligned} \right.$ | (Eq. 6) |
| --- | --- |

Where $t_{v,d}$ is the date of vaccination dose *d*, $\chi^{(d)}$ are the initial effectiveness of dose *d* (i.e., 14 days after vaccination) against the considered variant, and $w_{d}$ is the waning rate of vaccine protection for dose *d*. Estimates of vaccine effectiveness and waning rate were obtained from a large-scale test-negative case–control study [S6] and reported in Table S1. To reproduce at best the vaccines administered in Italy, we considered the effectiveness estimated for a COMIRNATY vaccine for the main schedule and a heterologous booster with Spikevax vaccine.

**Table S1.** Parameters for vaccine effectiveness and waning.

| Parameter | Unit | Dose | Value |
| --- | --- | --- | --- |
| Initial effectiveness (14 days after dose) | % | 1 | 0 |
|  |  | 2 | 76.3 |
|  |  | 3 | 76.5 |
| Waning rate | days^-1^ | 1 | 0 |
|  |  | 2 | 1/74.5 |
|  |  | 3 | 1/195.3 |

For individuals who had a previous infection with a different lineage, we assumed a cross-protection $\eta$=56% [S7] before he receives a complete primary vaccination cycle (first two doses), and a non-waning protection equal to the one conferred by the booster dose after completion of the primary cycle [S7]:

| $\chi_{j}\left( t \right)=\left\{ \begin{aligned} 1-\eta if {t<t}_{v,2}+14 \\ 1-\chi^{\left( 3 \right)} if t\geq t_{v,2}+14 \end{aligned} \right.$ | (Eq. 7) |
| --- | --- |

The model assigns a source of infection $k_{j}$ for all cases by choosing from either a generic source outside the household or from an infectious household member in H_j_, with probability proportional to the contribution of each source to the total force of infection $\lambda_{j}(T_{I,j})$ at the time $T_{I,j}$ at which j was infected. The overall likelihood of the observations given parameter set $\theta=(\alpha,\beta,a,b)$ and the assigned sources of infection $k_{j}$ is given by:

| $L\left( \theta, k_{j} \right)= \prod_{j} P_{j}Q_{j}$ | (Eq. 8) |
| --- | --- |

where

| $P_{j}= \left\{ \begin{aligned} \lambda_{j}^{o}\left( T_{I,j} \right)\mathrm{if}k_{j} is outside the household \\ \lambda_{j.i}^{h}\left( T_{I,j} \right) \mathrm{if} k_{j} is household member i \\ 1 \mathrm{if}j is uninfected \end{aligned} \right.$ | (Eq. 9) |
| --- | --- |

For infected individuals, $Q_{j}$is the probability that *j* has not been infected until $T_{I,j}$, namely $Q_{j}= e^{-\int_{0}^{T_{I,j}} \lambda_{j}\left( t \right)dt}$. For uninfected individuals, it is the probability that *j* has never been infected, $Q_{j}= e^{-\int_{0}^{\infty} \lambda_{j}\left( t \right)dt}$.

We estimated the unknown parameters $\theta$ and the source of infection $k_{j}$for all cases using a Monte Carlo Markov Chain (MCMC) procedure. At each step, all parameters in $\theta$ are updated using reversible normal jumps. Z=500 samples from the posterior distributions obtained by the MCMC for each of the K=100 samples were pooled together to obtain the final parameter distribution and the distribution of the sources of infection for each case.

The model for the inference of transmission links was implemented in C using GSL libraries (version 2.6) and compiled with GCC (version 4.2.1).

1. **Additional results of the baseline model**

*Statistics on reconstructed transmission links*

The mean per-household number of infections contracted from the general community was 1.15 (95%CrI 1.13 – 1.18). The mean number of secondary infections generated by a positive case was 0.56 (95%CrI 0.55 – 0.57). Table S2 shows how the model reconstructed transmission links within households with different numbers of cases.

**Table S2. Statistics for the model-based reconstruction of transmission links in households by number of SARS-CoV-2 cases.**

|  |  | |
| --- | --- | --- |
|  | ***Number*** | **%** |
| **Households with 2 SARS-CoV-2 cases** | ***5,579*** | **100** |
| - Both infected in the general community | *642*  *(540-770)* | 11.5  (9.7-13.8) |
| - One infected the other | *4,937*  *(4,809-5,039)* | 88.5  (86.2-90.3) |
|  |  |  |
| **Households with 3 SARS-Cov-2 cases** | ***2,080*** | **100** |
| - All infected in the general community | *18*  *(9-29)* | 0.8  (0.4-1.4) |
| - One transmission, 2 infected in the general community | *349*  *(300-410)* | 16.8  (14.4-19.7) |
| - Two transmissions, same infector (1 generation) | *838*  *(792-884)* | 40.3  (38.1-42.5) |
| - Two transmissions, different infectors (2 generations) | *875*  *(795-944)* | 42.0  (38.2-45.4) |
|  |  |  |
| **Households with 4 or more SARS-Cov-2 cases** | ***1,244*** | **100** |
| - All infected in the general community | *0*  *(0-2)* | 0  (0-0.2) |
| - One transmission | *174*  *(144-208)* | 14  (11.6-16.7) |
| - Two transmissions | *637*  *(604-670)* | 51.2  (48.6-53.9) |
| - Three or more transmissions | *432*  *(372-494)* | 34.8  (29.9-39.7) |

*Stability of the attributed source of infection*

For each case, we considered the distribution of the sources of infection attributed by the model and evaluated its stability. We categorized cases according to whether its source of infection was consistently (i.e., more than 75% of the times over the Z sampling of infector and K sampling of infectious dates) attributed to:

- the same household member;
- transmission within household but from different potential infectors;
- transmission in the general community.

The setting of transmission was uncertain (less than 75% consistency in attribution) in about 40% of cases (Figure S3). This generally happened when two or more cases in a household had close diagnosis dates, so that either could have been infected in the general community and then transmitted to the other, or both could have been infected in the general community, depending on the assigned dates of infection.

**Figure S3. Consistency in the attribution of the infector or the infector setting.** The stacked barchart represents the proportion of individuals that were consistently (more than 75% of the times across Z sampling of sources and K sampling of infectious dates) or inconsistently attributed to either category.

1. **Sensitivity Analyses**

We performed ten sensitivity analyses (SA) to test the robustness of model results against different model assumptions. The first SA (a) applies the baseline model to a subset of 380 households (1,127 cases in total) for which a case was genotyped as Omicron; the second (b) applies the baseline model to a subset of 1,148 households (2,770 cases in total) for which all individuals were unvaccinated; the third to sixth (c, d, e, f) impact on the main unknown of the data, i.e. the imputation of infection times. In particular, the third SA (c) assumes an incubation period equal to one previously estimated for Delta [S2] (mean: 4.5 days and standard deviation 2.1 days) to reassign the imputed infectious dates (baseline: Omicron variant with mean 3.5 days and standard deviation 1.2 days); the fourth (d) considers a distribution of the diagnostic delay for asymptomatic cases that is 50% longer than that for symptomatic cases, implemented by increasing the shape of the gamma distribution by 50% (mean: 7.58 days; standard deviation: 1.61 days); the fifth (e) is similar to d, but implemented by changing the scale of the gamma distribution by 50% (mean: 7.58 days; standard deviation: 1.97 days); Figure S4 shows a comparison of the diagnostic delay distributions used in SA d) and e). The sixth SA (f) allows false negatives test results when imputing infection dates; the seventh to tenth SA (g, h, i, j) consider slightly different model assumptions. In particular, the seventh (g) considers a reduced transmissibility for asymptomatic individuals; the eighth (h) considers a reduced transmissibility for vaccinated individuals, the ninth (i) assumes that any effort to quarantine positive cases would not impact the force of infection from outside the household (i.e., $q\left( t \right)=1$ for any value of t in equation 4) which corresponds to a 0% compliance to the policy; the tenth (j) evaluates the possibility that previous infection from other variants provides no residual natural immunity.

**Figure S4. Diagnostic delays distributions for asymptomatic individuals.** “Baseline” represents the distribution used in the main analysis, equal to the one estimated for asymptomatic cases; “Increased shape” is the distribution used in SA d); “Increased scale” is the one used in SA e).

1. *Only households with confirmed Omicron genotype*

In this sensitivity analysis we considered only the subset of households for which one case has been classified as Omicron after genotyping (380 households, 1,127 cases in total). Results are reported in Table S3.

**Table S3. Estimates for the intrinsic and realized household generation time and household serial intervals when considering only households with a confirmed Omicron genotype.**

| Intrinsic generation time | mean (95%CrI) [days] | | 6.20 (5.14-7.99) |
| --- | --- | --- | --- |
|  | shape mean (95%CrI) | | 2.51 (2.1-3.58) |
|  | scale mean (95%CrI) | | 2.53 (1.6-3.64) |
|  | Standard deviation of the mean distribution [days] | | 3.95 |
| Realized household generation time | mean (95%CrI) [days] | | 3.39 (3.36-3.4) |
| household Serial Interval | mean (95%CrI) [days] | | 2.27 (2.1-2.44) |
| Pre-symptomatic transmission | mean (95%CrI) [%] | 55.6 (51.8-59.2) | |

1. *Only households including unvaccinated individuals*

In this sensitivity analysis we considered only the subset of households where all individuals were unvaccinated (1,148 households; 2,770 cases in total). Results are reported in Table S4.

**Table S4. Estimates for the intrinsic and realized household generation time and household serial intervals when considering only households where all individuals were unvaccinated.**

| Intrinsic generation time | mean (95%CrI) [days] | | 7.65 (6.73-8.36) |
| --- | --- | --- | --- |
|  | shape mean (95%CrI) | | 2.08 (1.89-2.35) |
|  | scale mean (95%CrI) | | 3.69 (3.23-4.01) |
|  | Standard deviation of the mean distribution [days] | | 5.31 |
| Realized household generation time | mean (95%CrI) [days] | | 3.24 (3.23-3.26) |
| household Serial Interval | mean (95%CrI) [days] | | 1.89 (1.77-2.01) |
| Pre-symptomatic transmission | mean (95%CrI) [%] | 57.4 (54.8-60.2) | |

1. *Alternative distribution of the incubation period*

In this sensitivity analysis we considered an alternative distribution of the incubation period, previously estimated for the Delta variant, with mean 4.5 days (shape: 4.43; scale: 1.01) [S2]. Results are reported in Table S5.

**Table S5. Estimates for the intrinsic and realized household generation time and household serial intervals using an alternative distribution of the incubation period.**

| Intrinsic generation time | mean (95%CrI) [days] | 6.87 (6.15-8.12) |
| --- | --- | --- |
|  | shape mean (95%CrI) | 2.81 (2.09-3.49) |
|  | scale mean (95%CrI) | 2.53 (1.80-3.59) |
|  | Standard deviation of the mean distribution [days] | 4.15 |
| Realized household generation time | mean (95%CrI) [days] | 3.96 (3.96-3.97) |
| household Serial Interval | mean (95%CrI) [days] | 2.02 (1.96-2.08) |
| Pre-symptomatic transmission | mean (95%CrI) [%] | 58.8 (53.8-63.6) |

1. *Longer diagnostic delay for asymptomatic cases (increased shape of gamma distribution)*

In this sensitivity analysis we increased by 50% the diagnostic delay of asymptomatic cases. For asymptomatic cases, we considered a gamma function that has the same scale parameter as that estimated for symptomatic cases, and a shape parameter that is 1.5 times that estimated for symptomatic cases. Results are reported in Table S6.

**Table S6. Estimates for the intrinsic and realized household generation time and household serial intervals using a longer diagnostic delay for asymptomatic cases.**

| Intrinsic generation time | mean (95%CrI) [days] | 6.87 (5.94-8.07) |
| --- | --- | --- |
|  | shape mean (95%CrI) | 2.51 (2.01-3.27) |
|  | scale mean (95%CrI) | 2.83 (1.86-3.76) |
|  | Standard deviation of the mean distribution [days] | 4.39 |
| Realized household generation time | mean (95%CrI) [days] | 3.68 (3.67-3.68) |
| household Serial Interval | mean (95%CrI) [days] | 2.28 (2.22-2.33) |
| Pre-symptomatic transmission | mean (95%CrI) [%] | 55.3 (50.4-59.9) |

1. *Longer diagnostic delay for asymptomatic cases (increased scale of gamma distribution)*

In this sensitivity analysis we increased by 50% the diagnostic delay of asymptomatic cases. For asymptomatic cases, we considered a gamma function that has the same shape parameter as that estimated for symptomatic cases, and a scale parameter that is 1.5 times that estimated for symptomatic cases. Results are reported in Table S7.

**Table S7. Estimates for the intrinsic and realized household generation time and household serial intervals using a longer diagnostic delay for asymptomatic cases.**

| Intrinsic generation time | mean (95%CrI) [days] | 6.92 (6.01-8.07) |
| --- | --- | --- |
|  | shape mean (95%CrI) | 2.58 (2.02-3.3) |
|  | scale mean (95%CrI) | 2.77 (1.88-3.76) |
|  | Standard deviation of the mean distribution [days] | 4.36 |
| Realized household generation time | mean (95%CrI) [days] | 3.79 (3.79-3.8) |
| household Serial Interval | mean (95%CrI) [days] | 2.29 (2.23-2.34) |
| Pre-symptomatic transmission | mean (95%CrI) [%] | 55 (49.9-59.6) |

1. *False negative test allowed when imputing infection dates*

In this sensitivity analysis we allowed for false negative test results when imputing infection dates, i.e. we removed the factor $H({T_{I}-T}_{N})$ in Equations 1 and 2. Results are reported in Table S8.

**Table S8. Estimates for the intrinsic and realized household generation time and household serial intervals when allowing for false negative test when imputing infection dates.**

| Intrinsic generation time | mean (95%CrI) [days] | 6.68 (5.58-8.08) |
| --- | --- | --- |
|  | shape mean (95%CrI) | 2.42 (2.04-3.3) |
|  | scale mean (95%CrI) | 2.83 (1.8-3.75) |
|  | Standard deviation of the mean distribution [days] | 4.34 |
| Realized household generation time | mean (95%CrI) [days] | 3.4 (3.39-3.4) |
| household Serial Interval | mean (95%CrI) [days] | 2.23 (2.18-2.29) |
| Pre-symptomatic transmission | mean (95%CrI) [%] | 54 (49.4-58.1) |

1. *Reduced transmissibility for asymptomatic individuals*

In this sensitivity analysis, we consider a halved transmissibility for asymptomatic individuals [S8] by multiplying Equation 5 by an individual transmissibility $\varphi_{i}$, where $\varphi_{i}$ is 1 if *i* is symptomatic and 0.5 if asymptomatic. Results are reported in Table S9.

**Table S9. Estimates for the intrinsic and realized household generation time and household serial intervals using a halved transmissibility for asymptomatic individuals.**

|  |  |  | |
| --- | --- | --- | --- |
| Intrinsic generation time | mean (95%CrI) [days] | 6.66 (5.78-7.99) | |
|  | shape mean (95%CrI) | 2.55 (2.05-3.22) | |
|  | scale mean (95%CrI) | 2.65 (2.06-3.42) | |
|  | Standard deviation of the mean distribution [days] | 4.19 | |
| Realized household generation time | mean (95%CrI) [days] | 3.58 (3.57-3.58) | |
| household Serial Interval | mean (95%CrI) [days] | 2.36 (2.29-2.42) | |
| Pre-symptomatic transmission | mean (95%CrI) [%] | | 51.3 (46.4-55.6) |

1. *Reduced transmissibility for vaccinated individuals*

In this sensitivity analysis, we consider a halved transmissibility for vaccinated individuals [S8] by considering $\varphi_{i}$ = 0.5 for vaccinated and $\varphi_{i}$ = 1 for unvaccinated individuals. Results are reported in Table S10.

**Table S10. Estimates for the intrinsic and realized household generation time and household serial intervals using a halved transmissibility for vaccinated individuals.**

| Intrinsic generation time | mean (95%CrI) [days] | | 6.06 (5.5-6.62) |
| --- | --- | --- | --- |
|  | shape mean (95%CrI) | | 2.09 (1.9-2.3) |
|  | scale mean (95%CrI) | | 2.91 (2.45-3.39) |
|  | Standard deviation of the mean distribution [days] | | 4.20 |
| Realized household generation time | mean (95%CrI) [days] | | 3.46 (3.45-3.47) |
| household Serial Interval | mean (95%CrI) [days] | | 2.26 (2.22-2.32) |
| Pre-symptomatic transmission | mean (95%CrI) [%] | 53.0 (48.6-56.8) | |

1. *No protection from infection outside the household during quarantine*

In this sensitivity analysis, we assume that the imposed quarantine period after the first positive diagnosis would not impact the force of infection from outside the household (i.e., $q\left( t \right)=1$ for any value of t in Equation 4). Results are reported in Table S11.

**Table S11. Estimates for the intrinsic and realized household generation time and household serial intervals when assuming no protection from outside infection during the quarantine period.**

|  |  |  | |
| --- | --- | --- | --- |
| Intrinsic generation time | mean (95%CrI) [days] | 5.09 (4.37-5.78) | |
|  | shape mean (95%CrI) | 2.44 (2.09-4.21) | |
|  | scale mean (95%CrI) | 2.14 (1.06-2.7) | |
|  | Standard deviation of the mean distribution [days] | 3.30 | |
| Realized household generation time | mean (95%CrI) [days] | 3.39 (3.38-3.4) | |
| household Serial Interval | mean (95%CrI) [days] | 2.22 (2.16-2.28) | |
| Pre-symptomatic transmission | mean (95%CrI) [%] | | 53.6 (48.7-57.5) |

1. *No protection from previous infection*

In this sensitivity analysis, we assume that the previous infection would not impact the susceptibility to Omicron infection of an individual. Results are reported in Table S12.

**Table S12. Estimates for the intrinsic and realized household generation time and household serial intervals when assuming no protection from previous infection.**

|  |  |  | |
| --- | --- | --- | --- |
| Intrinsic generation time | mean (95%CrI) [days] | 6.88 (5.72-8.75) | |
|  | shape mean (95%CrI) | 2.37 (2-3.29) | |
|  | scale mean (95%CrI) | 2.99 (1.83-4.35) | |
|  | Standard deviation of the mean distribution [days] | 4.52 | |
| Realized household generation time | mean (95%CrI) [days] | 3.59 (3.55-3.59) | |
| household Serial Interval | mean (95%CrI) [days] | 2.38 (2.3-2.46) | |
| Pre-symptomatic transmission | mean (95%CrI) [%] | | 51.1 (45.3-55.7) |

1. **Epidemiological context**

In the Emilia Romagna region, where our study area is located, Omicron became dominant in the second half of December 2021 (Table S13), causing a large upsurge of cases that subsided by the end of January (Figure S5). A second smaller wave occurred since early March, likely due to the expansion of sublineage BA.2 (Table S13 and Figure S5). The selected study period allows to include a large majority of cases from the first Omicron wave while minimizing the risk of including multiple sublineages with different transmissibility in the data, which would require a significant complication of the adopted model.

**Table S13. Prevalence of the Omicron variant in genomic surveillance surveys conducted within the Emilia Romagna region, December 2021-April 2022.** Data from [S9].

| Relative prevalence | 2021 | | 2022 | | | | |
| --- | --- | --- | --- | --- | --- | --- | --- |
|  | Dec 6 | Dec 20 | Jan 3 | Jan 17 | Jan 31 | Mar 7 | Apr 4 |
| Omicron | 0.6% | 16.5% | 79.4% | 99% | 98.7% | 100% | 100% |
| of which BA.1 | NA^*^ | NA^*^ | NA^*^ | 97%^**^ | 97%^**^ | 41.7% | 6.4% |
| BA.2 | NA^*^ | NA^*^ | NA^*^ | 3%^**^ | 3%^**^ | 58.3% | 94.6% |

^*^ Sublineages were not genotyped on December 6, December 20 and January 3.

^**^ Sublineages were genotyped at the national level on January 17 and 31.

**Figure S5. Daily number of diagnosed cases in the province of Reggio Emilia between December 1, 2021 and April 15, 2022.** The study period of this work (January 1-31, 2022) is highlighted in light blue. Data from the Integrated Surveillance System of the Istituto Superiore di Sanità [S4, S5].

1. **Simulation of within-household transmission**

To illustrate the difference between the intrinsic and realized generation time, we built a simple computational model capable of generating infections within households while accounting for the possibility of acquiring infections from the community. First, we built a population of individuals distributed in households according to Italian statistics on household size [S10]. Then, we consider each individual to be subject to the force of infection defined in Equations 3-5, where I(z) is the number of symptomatic cases in the province of Reggio Emilia and parameters have been set to arbitrary values. In particular, we imposed $a=2.3$ and $b=3$ for the gamma-distribution of the generation time, corresponding to a mean intrinsic generation time of 6.9 days (standard deviation: 4.6 days). Every time an infection within the household occurs, we record the date of infection and the infector; for infections imported from the community we only recorded the date of infection. In this way, we can reconstruct the distribution of the realized generation times in household.

The model is implemented stochastically with a time step of 1 hour and applied to a population of 2,000 households for a duration of 60 days, where day 1 represented December 16, 2021, and day 60 represented February 14, 2022. After running the model, we filtered households with at least two cases, with less than 14 days elapsing between two consecutive infections, and with all diagnoses comprised in the period January 1-31, 2022. We obtained a mean realized generation time in households of 4.72 days (standard deviation: 0.09 days); 95%CrI of the mean: 4.54-4.90 days), i.e., significantly shorter than the intrinsic one, under parameters for transmissibility ($\alpha=5\cdot{10}^{-6}$ and $\beta=0.5$) that resulted in an attack rate of 70% and in a rate of 1.13 imported cases per household.

**References**

1. Brandal LT, MacDonald E, Veneti L, Ravlo T, Lange H, Naseer U, Feruglio S, Bragstad K, Hungnes O, Ødeskaug LE, Hagen F. Outbreak caused by the SARS-CoV-2 Omicron variant in Norway, November to December 2021. Eurosurveillance. 2021 Dec 16;26(50):2101147.
2. Manica M, Litvinova M, De Bellis A, Guzzetta G, Mancuso P, Vicentini M et al. Estimation of the incubation period and generation time of SARS-CoV-2 Alpha and Delta variants from contact tracing data. Arxiv preprint. Available at: <https://arxiv.org/abs/2203.07063>
3. G Guzzetta, C Minosse, R Pisapia, E Giombini, A Mammone, F Vairo, et al. Household transmission and disease transmissibility of a large HAV outbreak in Lazio, Italy, 2016–2017. Epidemics, 2019; 29, 100351
4. F Riccardo, M Ajelli, XD Andrianou, A Bella, M Del Manso, M Fabiani, et al.; COVID-19 working group. Epidemiological characteristics of COVID-19 cases and estimates of the reproductive numbers 1 month into the epidemic, Italy, 28 January to 31 March 2020. Euro Surveill. 2020. Dec;25(49):2000790. 10.2807/1560-7917.ES.2020.25.49.2000790
5. F Riccardo, G Guzzetta, A. Mateo Urdiales, M. Del Manso, XD Andrianou, A Bella, et al. COVID-19 response: effectiveness of weekly rapid risk assessments, Italy. Bulletin of the World Health Organization, 2022; 100(2): 161.
6. Andrews N, Stowe J, Kirsebom F, Toffa S, Rickeard T, Gallagher E, Gower C, Kall M, Groves N, O’Connell AM, Simons D. Covid-19 vaccine effectiveness against the omicron (B. 1.1. 529) variant. New England Journal of Medicine. 2022 Mar 2.
7. Altarawneh HN, Chemaitelly H, Hasan MR, Ayoub HH, Qassim S, AlMukdad S, Coyle P, Yassine HM, Al-Khatib HA, Benslimane FM, Al-Kanaani Z. Protection against the omicron variant from previous SARS-CoV-2 infection. New England Journal of Medicine. 2022 Feb 9.
8. D McEvoy, C McAloon, A Collins, et al Relative infectiousness of asymptomatic SARS-CoV-2 infected persons compared with symptomatic individuals: a rapid scoping review BMJ Open, 2021: 11:e042354.
9. Istituto Superiore di Sanità. Monitoraggio delle varianti del virus SARS-CoV-2 di interesse in sanità pubblica in Italia. Available at:

<https://www.epicentro.iss.it/coronavirus/sars-cov-2-monitoraggio-varianti-indagini-rapide>

1. ISTAT. Popolazione e famiglie. Available at <https://www.istat.it/it/popolazione-e-famiglie?dati> .
