# Supplementary material for: Intrinsic generation time of the SARS-CoV-2 Omicron variant: An observational study of household transmission
Source: Lancet Reg Health Eur. 2022 Jul 1;19:100446. doi: 10.1016/j.lanepe.2022.100446 (PMC9246701; doi:10.1016/j.lanepe.2022.100446)
Supplement: Supplementary file 2 [file mmc2.docx]

The following are members of the Reggio Emilia Covid-19 Working Group:

| **First name** | **Surname** | **Title** |
| --- | --- | --- |
| Emanuela | Bedeschi | MD |
| Cinzia | Perilli | MSc |
| Nadia | Montanari | MSc |
| Francesca Pia | Lionetti | MSc |
| Nicoletta | Patrignani | MSc |
| Letizia | Bartolini | MSc |
| Francesca | Roncaglia | PhD |
| Isabella | Bisceglia | MSc |
| Valeria | Cenacchi | PhD |
| Maria Barbara | Braghiroli | MA |
| Annamaria | Pezzarossi | MSC |
